# The Provision of Sanatoria

**Published:** 1912-07-06

**Authors:** 


### THE PROVISION OF SANATORIA.

The longer the complex and intricate ramifications of the Insurance Act are studied, the more certain does the student of it become that, to force on this legislation as a party question and to rush it through Parliament as a piece of party tactics, was the sort of blunder that is, if not worse than, certainly as bad as a crime.

Hitherto, the provisions of the Act relating to sanatorium benefit have received but slight criticism compared with that which has been showered upon most of the other sections of it. On closer scrutiny, however, it has been made apparent to several local authorities that this part of the Act also, like the rest of it, teems with difficulties, obscurities, and imperfections. It will be remembered that the Act provides for the outlay of £1,500,000 as a capital sum for the provision of sanatoria and other forms of treatment; and that the Local Government Board is made responsible for the distribution of this sum. Further, an annual sum of about one million sterling is allotted for the upkeep of these sanatoria. Unfortunately, this money has to come out of the funds provided by the working of the Act, and those local authorities who are keenly desirous of dealing effectively with tuberculosis within their boundaries are handicapped by uncertainty as to the amount of the grants that will be at their disposal.

To quote a concrete instance, the Kent County Council held a special meeting the other day to consider this very point. The suggestion of the Local Government Board that one sanatorium bed and one hospital bed should be provided per 5,000 of population means 400 beds for the whole county. As a commencement, the County Medical Officer proposed to provide 100 sanatorium beds and 105 hospital beds. It was calculated that this would cost the Council about £5,000 a year over and above the £21,000 which it was expected the Insurance Committee would be able to provide. Recently, however, an intimation has been received from the Insurance Commissioners that "as the Insurance Committee must incur considerable expense for home treatment they may find their funds insufficient to enable them to exercise the power of providing all the dependants of insured persons with sanatorium benefit." In other words, the County Council may proceed with their scheme if they like, but it is highly improbable that the Insurance Committee will be able to finance it. This is bad enough, but the next pronouncement makes it worse still. The communication proceeds that such dependants must be regarded as members of the general community, and it may fall upon the County Council to provide for their treatment for tuberculosis, as in the case of other insured persons. The exact import of this sentence seems a little doubtful, but it reads almost as if an attempt is to be made to compel local authorities to pay out of their own pockets for those very luxuries which have been trumpeted all over the kingdom as provided by the Chancellor's Act!

There are other local authorities besides those of Kent who have studied the sanatorium provisions of the Act. The Lord Mayors of Birmingham, Liverpool, and Manchester find "serious defects" in these provisions; and in a joint letter to the *Times* they assert that "the arrangements under the Act are so uncertain and so cumbrous that it is doubtful whether" local authorities will be able to carry out any national scheme for the prevention and treatment of the disease. They urge that sanatorium benefit should be withdrawn from the Insurance Act altogether, and that the money thus saved should be used as a grant to local authorities towards the maintenance of curative and preventive schemes. Local authorities who have at present to negotiate with the Insurance Committees do not feel certain of any definite annual grant, and many of them are alarmed at the liability they may have to accept. Considering the experience of the Kent County Council, just narrated, it is easy to sympathise with and to understand their alarm. The situation, the three Lord Mayors quite justly remark, would become still more difficult and costly to the ratepayer if an Insurance Committee should extend sanatorium benefit to dependants.

But, as we began by remarking, the Insurance Act was rushed through Parliament, crude and ill-considered as it was, by party tactics as parcel of the party game. Party politics dominates everything connected with it rather than considerations of national well-being and health. It is, therefore, most improbable that any suggested modification of its unworkable sections will receive any degree of favour, however admirable it may be, because all criticism is resented and all discussion stifled as far as possible. It is evident enough that, as far as sanatorium benefit is concerned, the ninepence for fourpence catchword is a dead-letter; or rather that the ratepayers will most likely incur the privilege of providing the ninepences without even receiving the fourpences as a set-off.

